# Validity and Reliability of the Attention-deficit/hyperactivity disorder (ADHD) in Epidemiological Studies: partial least squares-based confirmatory factor analysis

**Published:** 2019-08

**Authors:** Shila Hasanzadeh, Mohammad Asghari Jafarabadi, Homyoun Sadeghi-Bazargani

**Affiliations:** ^*a*^Department of Statistics and Epidemiology, Faculty of Health, Tabriz University of Medical Sciences, Tabriz, Iran.; ^*b*^Associate Professor in Statistics and Epidemiology, Department of Statistics and Epidemiology, Faculty of Health, Tabriz University of Medical Sciences, Tabriz, Iran.; ^*c*^Associate Professor in Epidemiology, Road Traffic Injury Research Center, Tabriz University of Medical Sciences, Tabriz, Iran.

## Abstract

**Background:**

Despite many types of research about tools of ADHD have been done over the past decade, lack of a Valid and Reliable tool is observed. The purpose of this study was to investigate the validity and reliability of ADHD questionnaire in epidemiological studies based on confirmatory factor analysis (PLS).

**Methods:**

The present study is a case-control study that was conducted on 456 male motorcyclists and research tool was ADHD questionnaire in 2013. A total of 30 items from experts and audience community were detected necessary. Content validity was verified using expert opinion and construct validity was verified by factor analysis. The results of the Partial Least Squares -based confirmatory factor analysis was done by SMARTPLS3 software. The Cv-communality index, whose positive values indicate the quality of the model and the Cv-redundancy was calculated.

**Results:**

According to expert’s opinion, content validity was confirmed. Model fit indicators (AVE> 0.5, SRMR= 0.08, NFI= 0.89) the adequacy of the model and the result structural validity Confirmed. Also, there was also a significant relationship between sub-scales and items. There was a direct, positive and significant relationship between the subscale ASS, CSS and DSS with ADHD. Cronbach s alpha (> 0.7) and combined reliability (> 0.7) confirmed the reliability of the questionnaire.

**Conclusions:**

The results showed the validity and reliability of the ADHD questionnaire in epidemiologic studies and various studies for review and generalizability suggested in other communities.

**Keywords:**

ADHD, CFA, Reliability and Validity, Traffic

